# 厄洛替尼100 mg/天治疗化疗后进展的晚期肺腺癌的临床研究

**DOI:** 10.3779/j.issn.1009-3419.2011.06.09

**Published:** 2011-06-20

**Authors:** 春雷 施, 宝惠 韩, 爱琴 顾, 丽纹 熊, 洁 沈

**Affiliations:** 200030 上海，上海交通大学附属胸科医院肺内科 Department of Pulmonary Medicine, Shanghai Chest Hospital, Shanghai Jiaotong University, Shanghai 200030, China

**Keywords:** 肺肿瘤, 厄洛替尼, 靶向治疗, 表皮生长因子受体酪氨酸激酶抑制剂, Lung neoplasms, Erlotinib, Target therapy, EGFR-TKI

## Abstract

**背景与目的:**

厄洛替尼是一种表皮生长因子受体酪氨酸激酶抑制剂，标准治疗剂量为150 mg/天。部分患者因毒副反应导致减量或终止治疗。本研究对厄洛替尼100 mg治疗晚期化疗失败的肺腺癌患者的疗效和安全性进行观察总结。

**方法:**

40例经病理组织学或细胞学确诊为腺癌的晚期非小细胞肺癌患者，无吸烟史，给予厄洛替尼100 mg/次/天，直至疾病进展或不良反应不能耐受为止，观察疗效、无疾病进展时间、不良反应及与临床特征之间的关系。

**结果:**

40例患者中，11例部分缓解，14例稳定，15例进展，总缓解率为27.5%，疾病控制率为62.5%。中位无疾病进展时间（progression free survival, PFS）为5.5个月。外周血检测*EGFR*突变共15例，占37.5%，总缓解率为33.3%，疾病控制率为73.3%，EGFR有突变者的PFS为8.4个月。ECOG评分0分-1分患者中位PFS为6.1个月，ECOG评分2分患者中位PFS为3.1个月，两组差异有统计学意义（*P*=0.043）。最常见的不良反应是皮疹和腹泻，对症处理后缓解。

**结论:**

厄洛替尼100 mg治疗晚期肺腺癌有一定的疗效，安全性高，对不吸烟的腺癌患者、体力状况好、伴EGFR突变的患者获益更多。

## Abstract (en)

**Background and objective:**

Erlotinib is a epidermal growth factor receptor-tyrosine kinase inhibitor (EGFR-TKI). The standard dose is 150 mg/d. But some patients have to reduce the dose or stop treatment due to its side effects. The aim of this study is to evaluate the efficacy and safety of erlotinib 100 mg on patients with advanced adenocarcinoma lung cancer previously failed to chemotherapy.

**Methods:**

Forty adenocacinoma patients with histologically confirmed diagnosis were enrolled to be given erlotinib 100 mg/d until disease progression or occurrence of intolerable toxicity. Relationships between therapeutic effects of erlotinib, progression-free survival, side effects and the clinical characters were analyzed.

**Results:**

Among 40 patients, 11 got partial response, 14 had a stable disease and 15 had a progressive disease. The total response rate was 27.5%, and the disease control rate was 62.5%. The median progression-free survival was 5.5 months. Fifteen patients were positive for *EGFR* mutation according to the result of peripheral blood test. The response rate and disease control rate in 15 EGFR-mutation-positive patients were 33.3% and 73.3%, respectively, and the PFS was 8.4 months for 15 patients. The patients with ECOG 0-1 had a significant better PFS than those with ECOG 2 (6.1 *vs* 3.1 months) (*P*=0.043). The most frequent toxicities of erlotinib were rash and diarrhea which were easily controlled.

**Conclusion:**

Erlotinib 100 mg is effective and safe in treatment of patients with advanced adenocarcinoma, especially for non-smoking patients, patients with good performance status and EGFR-mutation-positive patients.

肺癌目前仍然是全球发病率和死亡率最高的恶性肿瘤。非小细胞肺癌（non-small cell lung cancer, NSCLC）约占所有肺癌的80%，多数病人死于复发或转移，Ⅲ期和Ⅳ期NSCLC的5年生存率低于5%^[1]^。晚期NSCLC的一线治疗的标准化疗方案是含铂的两药联合方案。临床研究^[2]^显示一线治疗后约有30%-40%的患者因复发转移转入二线治疗。目前二线化疗的标准方案是多西紫杉醇单药75 mg/m^2^三周方案，但有效率仅7%^[3]^。近年来，随着对肿瘤生物学研究的进展，分子靶向治疗逐渐成为NSCLC的重要治疗模式，临床研究的结果也带来很多亮点。厄洛替尼是一种表皮生长因子受体酪氨酸激酶抑制剂（epidermal growth factor receptor-tyrosine kinase inhibitor, EGFR-TKI），通过抑制表皮生长因子受体激酶的自磷酸化反应抑制信号转导，从而抑制肿瘤细胞的增殖和活化^[4]^。目前已经被美国NCCN指南列入一线、二线和三线治疗的标准治疗方案之一，标准的治疗剂量为150 mg/天。但其不良反应也不容忽视，如皮疹、腹泻、肝功能损害和间质性肺炎等，导致患者减量或终止治疗。部分患者在减量至100 mg/天治疗后依然观察到稳定的疗效，但毒副反应明显减轻。本文对我院应用厄洛替尼100 mg/天二线治疗的40例晚期肺腺癌患者进行观察，并检测外周血*EGFR*突变情况，对厄洛替尼100 mg/天的疗效、无疾病进展时间（progression free survival, PFS）和安全性进行观察总结。

## 1 材料与方法

### 1.1 入组标准

入组患者来自上海交通大学附属胸科医院，2009年4月-2009年6月经病理组织学或细胞学证实为腺癌的Ⅲb期或Ⅳ期NSCLC患者，无吸烟史、预计生存时间大于3个月；既往至少接受过1个疗程的标准化疗后复发转移；有可测量或可评价的肿瘤病灶；治疗期间不能使用其他抗肿瘤药物治疗；既往未使用过EGFR-TKI。

### 1.2 临床资料

共40例NSCLC患者，经病理组织学或细胞学证实为腺癌Ⅲb期或Ⅳ期。均无吸烟史。男性11例，女性29例，年龄28岁-72岁，中位年龄54岁。患者既往接受过1个-6个疗程的化疗失败，之前治疗方案为三代新药联合的含铂方案。

### 1.3 *EGFR*基因外显子19缺失和外显子21 L858R突变检测

采用酶切富集PCR方法，进行*EGFR*基因外显子19缺失及外显子21 L858R点突变的检测。EGFR外显子19的外引物为5’-atcccagaaggtgagaaagataaaattc-3’和5’-cctgag-gttcagagccatgga-3’，内引物为5’-gataaaattcccgtcgctatc-3’和5’-acccccacagcaaagcag-3’。EGFR外显子21的外引物为5’-cagc-caggaacgtactggtga-3’和5’-tccctggtgtcaggaaaatgct-3’，内引物为5’-cgcagcatgtcaagatcacagat-3’和5’-tccctggtgtcaggaaaatgct-3’，分别扩增EGFR外显子19和21。PCR反应条件为：94 ℃变性30 s，60 ℃退火30 s，72 ℃延伸30 s，循环次数35。第一轮反应产物分别采用限制性内切酶*Mse*Ⅰ和*Msc*Ⅰ富集突变基因，选择性酶切第一轮PCR扩增产物中EGFR外显子19和21的野生型序列，行第二轮PCR扩增，PCR反应条件同前。取第二轮PCR扩增产物3 μL-6 μL行12%聚丙烯酰胺凝胶电泳，硝酸银染色，放入凝胶成像系统，检测外显子19和外显子2l的突变情况。

### 1.4 治疗方法

口服厄洛替尼100 mg/天（商品名：特罗凯，罗氏公司），服用至疾病进展或出现不可耐受的不良反应。每月进行疗效和不良反应评估。所有患者签署知情同意书，并经伦理委员会批准。

### 1.5 疗效和不良反应评价

服药前后均行胸部CT扫描评价病灶大小，按照实体瘤疗效评价标准（RECIST）作为近期疗效评定标准，分为完全缓解（complete response, CR）、部分缓解（partial response, PR）、稳定（stable disease, SD）和进展（progression disease, PD），计算客观有效率（response rate, RR）和疾病控制率（disease control rate, DCR）。采用ECOG评分进行全身体力状况（perfor-mance status, PS）评估。药物不良反应按照NIC-CTC第3版评价毒性反应。PFS的起点事件为患者开始服用厄洛替尼，终点事件为疾病进展。随访截止日期为2010年8月1日。

### 1.6 统计学方法

数据采用SPSS 13.0统计软件进行处理，计数资料采用卡方检验进行比较分析。生存资料采用*Kaplan-Meier*法分析。*P* < 0.05为差异有统计学意义。

## 2 结果

### 2.1 近期疗效评价

全组共40例患者，至少完成6周药物治疗并接受疗效评价。结果显示厄洛替尼治疗后PR 11例，SD 14例，PD 15例。RR为27.5%（11/40），DCR为62.5%（25/40）。

### 2.2 外周血检测结果

所有患者都接受外周血EGFR检测，结果显示EGFR突变共15例（37.5%）。其中，外显子19突变共3例（7.5%），外显子21突变共13例（32.5%）。外显子19和21均有突变者1例。女性突变10例，男性突变5例。

### 2.3 不良反应

与药物相关的不良反应有皮疹、腹泻、乏力、恶心。皮疹0度6例，1度21例，2度13例，未见3度以上皮疹。腹泻0度9例，1度20例，2度11例，未见3度以上腹泻。有1例患者服药1个月因肝功能损害达3度停药，经治疗2周后恢复继续用药。1例患者因血小板下降达3度停药，停药2周后未恢复，终止治疗。

### 2.4 有效率、PFS和患者临床特征因素间的关系（表 1）

**1:** 100 mg厄洛替尼治疗晚期腺癌患者的临床特征和疗效、生存的关系 The efficacy of patients with treatment of erlotinib 100 mg in advanced adenocarcinoma

Varial/Category	Entire sample (*n*=40)	RR (%)	DCR (%)	PFS (month)	*P*
Age (year)					0.660
≥60	18	27.78 (5/18)	55.56(10/18)	6.2	
< 60	22	27.27 (6/22)	68.18(15/22)	5.2	
Gender					0.204
Male	11	27.27 (3/11)	54.55 (6/11)	2.4	
Female	29	27.59 (8/29)	65.52(19/29)	7.3	
EGFR mutation					0.058
Positive	15	33.33 (5/15)	73.33(11/15)	8.4	
Negative	25	20.00 (5/25)	52.00(13/25)	5.2	
19 positive	3	100.00 (3/3)	100.00 (3/3)	8.4	
21 positive	13	23.08 (3门3)	69.23 (9/13)	10.3	
PS score					0.043
0-1	31	32.25 (10/31)	70.97 (22/31)	6.2	
2	7	14.29 (1/7)	42.86 (3/7)	3.1	
Rash					0.050
Grade 1	21	23.80(5/21)	57.14(12/21)	5.8	
Grade 2	13	46.15(6/13)	100.00(13/13)	6.5	
RR: response rate; DCR: diease control rate; PFS: progression-free survival.					

所有突变患者缓解率和疾病控制率分别为33.33%和73.33%，而无突变患者缓解率和疾病控制率分别为20.00%和52.00%。其中21外显子突变者缓解率为23.08%，疾病控制率为69.23%。19缺失者缓解率和疾病控制率均为100.00%。

从缓解率和疾病控制率与临床特征间的关系来看，本组患者均为腺癌不吸烟，在年龄、性别、ECOG评分上未见缓解率和疾病控制率的差异。从皮疹发生情况来看，皮疹发生1度的缓解率和疾病控制率分别为23.80%和57.14%，2度皮疹的缓解率和疾病控制率分别为46.15%和100.00%。6例无皮疹患者疗效均为PD。

随访至2010年8月1日，服用厄洛替尼后35例患者出现进展，中位PFS为5.5个月。其中，ECOG评分0分-1分者中位PFS为6.2个月，ECOG评分2分者中位PFS为3.1个月，两组差异有统计学意义（*P*=0.043）（图 1）。EGFR有突变和无突变患者的中位PFS分别是8.4个月和5.2个月，差异无统计学意义（*P*=0.058）（图 2）。年龄、性别以及皮疹发生两组中位PFS都未见差异。

**1:**
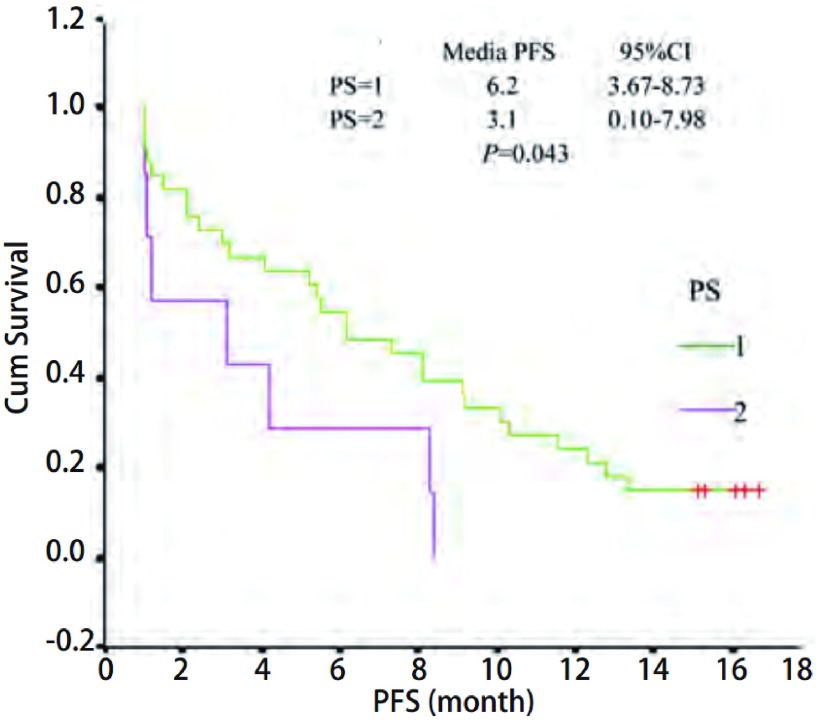
不同体力状况的PFS比较 PFS curve of PS

**2:**
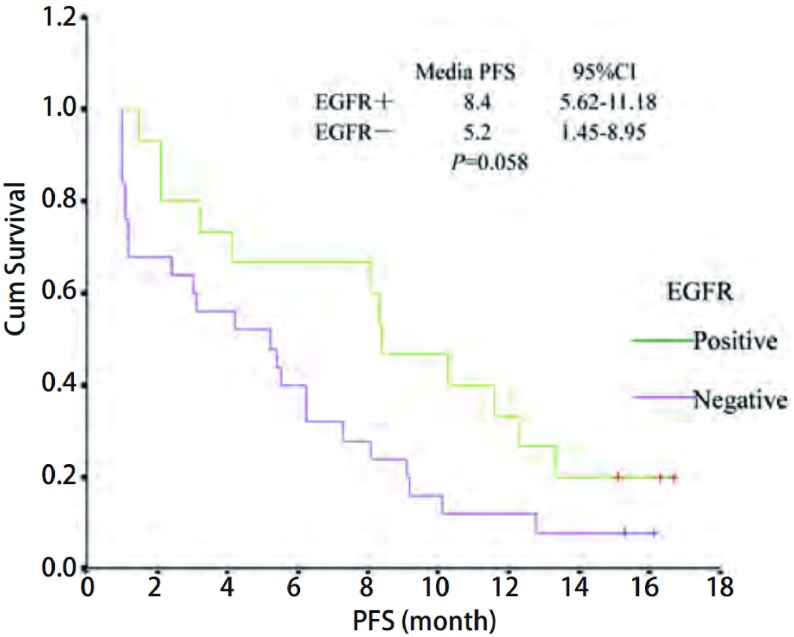
*EGFR*突变状况的PFS比较 PFS curve of *EGFR* mutation

## 3 讨论

厄洛替尼是一种EGFR-TKI，可与ATP竞争结合表皮生长因子受体（HER1/EGFR）酪氨酸激酶的细胞内催化区，抑制磷酸化并阻断下游信号传导从而抑制肿瘤的生长^[5]^。BR.21研究^[6]^结果显示，与安慰剂相比厄洛替尼治疗组的中位生存期为6.7个月，1年生存率31%，RR为8.9%，DCR为44%，亚裔患者的DCR达到78%。厄洛替尼能改善肿瘤相关症状，延长中位生存期。这种生存受益见于多个亚组，包括吸烟状态、性别、组织学类型、体能状态。2004年美国FDA批准该药用于二线或三线治疗局部晚期或Ⅳ期NSCLC，推荐剂量为150 mg/天。

TRIBUTE研究^[7]^结果显示不吸烟的患者中紫杉醇/卡铂联合厄洛替尼较单纯化疗组生存期有明显延长（22.5个月*vs* 10.1个月，*P*=0.01）。TRUST研究结果显示^[8]^近80%的Ⅲb期和Ⅳ期患者可从厄洛替尼二线治疗中获益，中位PFS达28周，1年生存率达63%。SATURN研究^[9]^的结果显示对于亚裔人群，厄洛替尼组总生存时间与安慰剂相比有获益趋势（20.8个月*vs* 15.2个月），有*EGFR*突变患者化疗疗效稳定后给予维持治疗中位生存期达22.1个月。西班牙的研究^[10]^结果显示，晚期NSCLC伴*EGFR*突变者厄洛替尼单药治疗的有效率达73%，TTP为12个月，中位生存期为24个月，其中EGFR第19外显子缺失和第21外显子突变患者的生存期分别为27个月和16个月，有效率和生存期均高于常规的化疗。基于这些研究，2009版指南推荐如明确有EGFR活化突变或基因扩增且患者无吸烟史，可考虑厄洛替尼±化疗方案。2011年指南更新为对于*EGFR*突变阳性的患者，可予一线治疗厄洛替尼或吉非替尼。

多项临床试验证实了厄洛替尼在肺癌治疗中的疗效，但即使是靶向治疗也存在一定的不良反应，部分患者在治疗过程中出现皮疹、腹泻、肝功能异常和间质性肺炎，往往需要减量或终止治疗。厄洛替尼Ⅰ期临床试验评价了每日和每周口服厄洛替尼治疗晚期肿瘤患者的安全性，结果发现在剂量达到200 mg/天时，出现剂量限制性毒性，主要为腹泻和皮疹，推荐进入Ⅱ期临床试验的剂量为150 mg/天^[11]^。Perez-Soler等^[12]^探讨了厄洛替尼治疗曾化疗过的晚期NSCLC的疗效，入组对象为EGFR表达阳性的患者，口服厄洛替尼150 mg/天至疾病进展。不良反应评定结果显示主要为皮疹和腹泻，发生率分别为67%和56%，与Ⅰ期临床试验的结果相似。SATURN研究^[9]^显示有2.8%的患者因治疗相关不良反应而终止治疗。BR.21研究^[13]^中有5例患者因为毒性终止治疗。TRUST研究显示^[8]^厄洛替尼相关严重不良反应的发生率为3%-5%，82%的患者出现皮疹，严重皮疹的发生率为10%。这些发生严重不良反应但治疗有效的患者，临床上会予以减量至100 mg继续治疗，依然可以观察到稳定的疗效。如果在这些获益人群中厄洛替尼100 mg的疗效与150 mg相当，那无论从临床应用还是经济学角度都有一定的实践意义。

本研究厄洛替尼剂量为100 mg/天，入组患者进行了选择，均为腺癌不吸烟患者。总缓解率为27.5%（11/40），疾病控制率为62.5%（25/40）。该结果高于BR.21的研究结果，与TRUST的研究结果相似，而其治疗剂量为100 mg。ECOG评分0分-1分的疗效优于2分者，但未见统计学差异，而性别年龄亦未见疗效差异。ECOG评分0分-1分者中位PFS为6.2个月，ECOG评分2分者中位PFS为3.1个月，两组差异有统计学意义（*P*=0.043）。外周血EGFR检测结果显示突变率37.5%，其中19外显子缺失率为7.5%，21外显子突变率为32.5%。所有突变患者缓解率和疾病控制率分别为33.33%和73.33%，其中21点突变者缓解率23.08%，疾病控制率69.23%。19缺失者缓解率和疾病控制率均为100%。EGFR有无突变患者的中位PFS分别是8.4个月和5.2个月，差异无统计学意义（*P*=0.058），与既往研究^[6, 7, 14]^结果相似。

临床研究显示厄洛替尼的常见不良反应为皮疹和腹泻，大多为轻度到中度，对症处理可缓解，本研究结果显示，皮疹和腹泻均为1度-2度，未见3度皮疹和腹泻。有2例患者因不良反应不可耐受而影响治疗，1例患者服药1个月因肝功能损害达3度暂停用药，2周后恢复继续用药。另1例患者因血小板下降达3度终止治疗。可见厄洛替尼100 mg治疗剂量也有一定的毒副反应，但与150 mg相比疗效相当，毒副反应更低。从本组研究来看，对于不吸烟腺癌患者，厄洛替尼100 mg与150 mg初步可见到相同的疗效，毒副反应低，并有一定的经济学效益。但本研究病例数较少，入组患者经过了选择，且外周血检测方法的准确率也未作验证，其结果有待进一步扩大样本并进行随机对照研究验证。

晚期NSCLC的治疗目的是延长患者生存期，改善患者的生活质量。EGFR-TKI制剂在二线、三线治疗上能达到与标准化疗同等的疗效，但毒副反应大大低于化疗，对患者生活质量的影响降到最低。本研究的结果也证实了EGFR-TKI制剂的优势。在亚裔、腺癌、不吸烟、全身状况较好的患者中100 mg也能看到初步相似的疗效，具有一定的经济学价值。而外周血*EGFR*突变对于EGFR-TKI制剂选择的指导意义仍有待临床试验验证。

本研究结果提示厄洛替尼对于亚洲不吸烟且为腺癌的晚期NSCLC患者具有良好的疗效，且毒副反应低。对部分患者100 mg的剂量同样能达到相似的疗效。随着分子标记物检测研究的进展，*EGFR*基因的表达、突变状况、*K-RAS*基因突变检测都将对靶向药物的选择提供更可靠的依据^[13, 15, 16]^。相信不久的将来分子标记物检测结果必定为临床靶向治疗的选择提供更有价值的依据。
